# Red Spectral Shift in Sensitive Colorimetric Detection of Tuberculosis by ESAT-6 Antigen-Antibody Complex: a New Strategy with Gold Nanoparticle

**DOI:** 10.1186/s11671-018-2753-5

**Published:** 2018-10-23

**Authors:** Fu-an Wang, Thangavel Lakshmipriya, Subash C. B. Gopinath

**Affiliations:** 1 Pingdingshan University Medical School, Pingdingshan City, 467000 Henan China; 2 Centre of Innovative Nanostructure & Nanodevices, Universiti Teknologi PETRONAS, 32610 Bandar Seri Iskandar, Perak Darul Ridzuan Malaysia; 3 School of Bioprocess Engineering, Universiti Malaysia Perlis, 02600 Arau, Perlis Malaysia; 4 Institute of Nano Electronic Engineering, Universiti Malaysia Perlis, 01000 Kangar, Perlis Malaysia

**Keywords:** Tuberculosis, ESAT-6 antigen, Colorimetric assay, Antibody, Gold nanoparticle

## Abstract

Tuberculosis (TB) is a highly contagious life-threatening disease caused by the bacterial pathogen *Mycobacterium tuberculosis*. ESAT-6, an abundant early secretory antigenic target protein by *M*. *tuberculosis*, found to play a vital role in virulence. Developing a friendly method for the detection of ESAT-6 at the lower concentration facilitates to treat TB at an earlier stage and helps to control the spreading of disease. Herein, a new single-step approach was designed and was done by pre-mixing ESAT-6 and antibody before being added to the gold nanoparticle (GNP) followed by the salt-induced aggregation. We could attain the detection limit of 1.25 pM, showing the aggregation of GNP and the red spectral shift. Further, a higher specificity was demonstrated with the lack of electrostatic biofouling by ESAT-6 on GNP and retained the dispersed GNP in the presence of 10-kDa culture filtrate protein from *M. tuberculosis*. The required precise antibody concentration for this assay was found to be 60 nM. The increment in the antibody concentration from 75 nM drastically diminishes the sensitivity to ~ 680-fold, due to the crowding effect. With this assay, we attested the suitability of colorimetric assay for efficiently detecting the smaller-sized protein.

## Background

Tuberculosis (TB) is caused by *Mycobacterium tuberculosis*, one of the leading death-causing diseases in human, and initially, it attacks the lung. As a consequence, it spreads over other parts of the body such as the kidney, spine, and brain and becomes more severe when the immunity lowers. To save people with precautionary action, it is mandatory to identify and treat TB at the latent stage. Under this stage, the patient has been infected by *M. tuberculosis*; however, the pathogen is not active. There are various methods that have been used to detect TB at a latent stage, which includes TB skin test and interferon-gamma test. In most instances, TB skin test alone is not able to detect the active TB infection, and the patient needs to be confirmed by other supporting tests, such as chest X-ray, sputum cytology, and sputum culture [1]. But these tests need harder laboratory steps and consume several months to complete; therefore, developing an easier and efficient method for detecting TB at an earlier stage is mandatory.

Gold nanoparticle (GNP)-based colorimetric assay shows a great attention in the field of biosensor due to its unique physical and chemical characteristics, such as higher extinction coefficient at the visible wavelengths and alterable surface plasmon resonance. Monodispersed GNP solution displays high extinction coefficients and shows the spectrum in the visible region. If GNPs have the proper space among them, they will appear to be red-colored solutions and will turn to blue when they are getting aggregated under the available condition of divalent ions [2]. In the presence of the appropriate ions, the spaces among GNPs will be filled, reach the stage of aggregation, and display the spectral shift towards the visible wavelength called ‘red shift’. By taking the advantage of this spectral shift, a lot of colorimetric assays have been generated to detect the diseases, such as HIV and influenza [3, 4]. These kind of assays are relying on single strand oligonucleotide sequences, and aptamer has been widely used as a suitable molecule for detecting the target [5, 6]. Single-strand DNA or RNA sequences can bind on the surface of GNP through the coordination between gold and nitrogen atoms in the DNA bases [7–9]. The modified GNPs with oligonucleotide are stable under higher salt conditions. When the analyte is available, oligonucleotide will be separated from the gold and gets aggregated in the presence of divalent ions then display the blue solution. The demonstrated GNP assays are cheaper, consuming lesser time for detection, easier to functionalize, and shows the visual detection with a good sensitivity [10–14]. Due to these positive attitudes, GNP-based colorimetric assays have been extended for the detection of smaller molecules including DNA, RNA, protein, whole cell, and metal ions. Several aptamers were available to detect these analytes by using the simplified aptamer GNP-based colorimetric assays, showing the red spectral shifts [15–19].

**Fig. 1:**
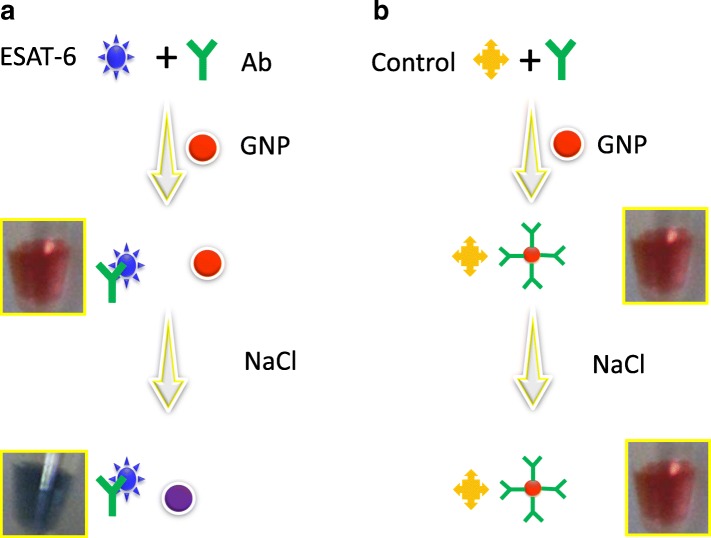
Schematic representation for ESAT-6 detection by a single-step pre-complexed antibody-based colorimetric assay. **a** Strategy for anti-ESAT-6 antibody pre-complexed ESAT-6. **b** Strategy for anti-ESAT-6 antibody pre-complexed CFP-10 (control experiment)

Even though colorimetric assays have been well documented with the aptamer, DNA or RNA, they also have negative impacts in some instances due to their poor performance with longer sequences and failure with the probe and target interactive analysis [20–22]. These might be due to the higher charges on the biomolecules that make a stronger interaction with GNP surface electrostatically, ultimately making the target molecule unable to release the attached probe molecule on the GNP surface. To overcome this hurdle, here, we introduced a single-step antibody-based colorimetric assay to detect the ESAT-6 protein. ESAT-6 protein is the early secretary protein, identified as the important antigen in tuberculosis [23–25]. Diagnosing ESAT-6 protein at an earlier stage, it is mandatory to treat the disease and avoid spreading. Figure 1a, b illustrates the strategy designed to detect ESAT-6 by its antibody using GNP-based colorimetric red spectral shift assay. The following are the steps involved in this detection method: (i) anti-ESAT-6 polyclonal antibody was pre-mixed with ESAT-6/CFP-10 antigen, (ii) GNP was added to this solution, (iii) NaCl was added, and (iv) visual detection and red spectral shift analysis by UV-spectroscopy was performed. With these steps, we performed the critical analysis to elucidate the charge-based interaction between GNP and the complexed proteins and concluded the suitability of pre-mixing antibody-antigen for the colorimetric assays. For the control experiment, the culture filtrate protein-10 (CFB-10) was used instead of ESAT-6 (Fig. 1).

## Methods

### Reagents and Biomolecules

Early secretory antigenic target (ESAT-6) and 10 kDa culture filtrate protein (CFP-10) were procured from Sino Biological Inc. (Beijing, China). Anti-ESAT-6 was purchased from Santa Cruz Biotechnology (USA). The gold nanoparticle with a diameter of 15 nm was obtained from Sigma Aldrich (USA). Deionized water produced by RO deionization system (18.3 MΩ·cm SASTEC (M) Sdn. Bhd) was availed for the entire experiment.

### Optimization of Divalent Ions to Aggregate GNP

For the detection of ESAT-6 in the current study, divalent ions (NaCl) were used for visualizing the aggregation of GNP. To optimize the suitable condition with NaCl, different concentrations (final concentrations to be 50 to 800 mM) were added to the constant volume (20 μl) of GNP [one optical density (O.D.)]. After 15 min, the color changes were photographed using a SONY digital camera. The spectral shifts with these changes were monitored by the Nanophotometer.

### ESAT-6 Biofouling on GNP Surface: Validation of Specificity

To validate the non-specific (biofouling) binding of ESAT-6 on GNP surface, different concentrations (final concentrations to be 1.5 to 100 nM) were added to 20 μl of GNP. After 30 min, the optimal concentration of NaCl was added to each tube and then the color changes and spectral shifts were observed as followed in the above experiment. Similarly, the biofouling was also tested with CFP-10.

### Optimization of Mandatory Anti-ESAT-6 Antibody Concentration on GNP Surface

To optimize the necessary concentration of anti-ESAT-6 antibody to evaluate the current experiments, different concentrations of anti-ESAT-6 antibody (final concentrations to be 30 to 500 nM) were mixed independently with 20 μl of GNP. After 30 min of incubation, the optimal volume of NaCl was added to each tube, photographs were taken, and spectral changes were noted after 15 min.

### ESAT-6 Detection by the Antibody-based Colorimetric Red Spectral Shift

To develop the colorimetric detection of ESAT-6 by antibody-based colorimetric red shift spectral changes, 1 μl of 1 μM ESAT-6 (final volume will be 500 nM) was initially mixed with 1 μl of optimal anti-ESAT-6 antibody concentration. After 30 min of incubation, 20 μl of GNP was added to each tube and then waited for 30 min. Then, the optimal NaCl concentration was added, and the spectral changes were measured under the wavelength from 400 to 800 nm. Similarly, the other concentrations of ESAT-6 (0 to 500 nM) were titrated with the same optimized anti-ESAT-6 antibody concentration. To check the limit of detection, ESAT-6 was tested from the lowest picomolar (from 1.25 pM) until the nanomolar (500 nM) order. The concentrations of other molecules (anti-ESAT-6 antibody, GNP and NaCl) were kept constant.

## Results and Discussion

The antibody-based colorimetric assay showing the spectral changes as a red-shift was followed here to detect ESAT-6. Figure 1a shows the schematic representation of single-step antibody-based colorimetric assay; in the presence of ESAT-6, the color change in GNP solution is expected to be blue with red spectral shift upon losing the stability of GNP under a high concentration of a divalent ion, NaCl, whereas, in the absence of ESAT-6, GNP is stable due to the attachment of the anti-ESAT-6 antibody on the GNP surface and leads to retaining its original red color even at a high concentration of NaCl. This strategy was confirmed by using the non-specific CFP-10 against anti-ESAT-6 antibody and it supposed to display a red color GNP solution (Fig. 1b). Since CFB-10 is also the major protein in causing tuberculosis, here, we used as the control protein [26]. Generally, for this kind of GNP-based assay, 13 to 15 nm-sized GNP is well suited to reach the higher sensitivity [27], and increasing GNP size is not suitable to achieve the good limit of detection. It is due to the reason that the increasing size of GNP needs a higher amount of antibodies to bind on the GNP surface. If a higher amount of antibody binds on the surface, it leads to the false negative result. Here, we desired to use 15 nm GNP and attained the maximal sensitivity to sub-picomolar level.

### Divalent Ion Optimization for Controlled GNP Aggregation

**Fig. 2:**
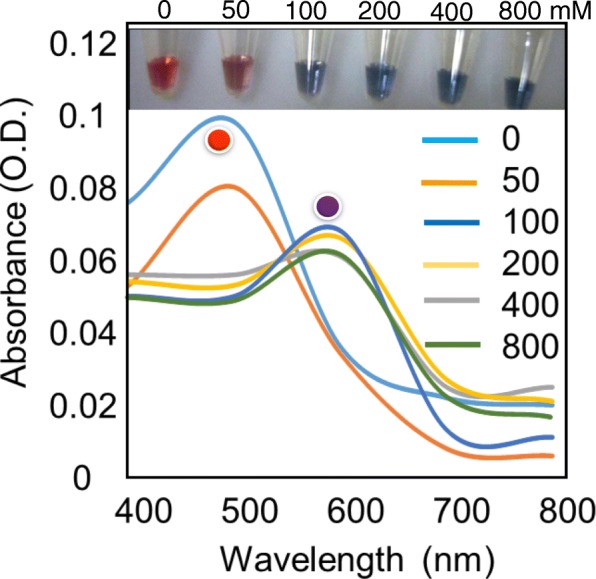
Optimization of divalent ions. From 0 to 800 mM of NaCl were mixed with constant GNP (1 O.D.). After 15 min, scanned at the wavelength from 400 to 800 nm. Photographs were taken by the digital camera as in figure inset. The spectrum and color changes are indicated by the aggregation of GNP with 100 mM NaCl. Peaks were indicated with the respective colored spheres

For this detection optimization, we used NaCl for the aggregation of GNP. When we screened for the necessary NaCl concentration as shown in Fig. 2, different concentrations of NaCl were added to the GNP, the color of the solution started to appear as blue, indicating the aggregation with 100 mM of NaCl. And also from the spectrum, it was clearly seen that only with the aggregated GNP (NaCl concentrations from 100 to 800 mM), there is a red-shift at the wavelength ~ 600 nm, whereas the dispersed GNP (NaCl concentrations from 0 to 50 mM) still maintain its spectrum at ~ 500 nm. However, there is an insignificant peak spectral change with 50 mM of NaCl. From these results, it was concluded that we need at least 100 mM of NaCl for the aggregation of GNP, but in order to get the enhanced sensitivity, we used a higher concentration (800 mM) of NaCl for further experiments. Previously, the similar range of NaCl for the aggregation of GNP was shown by Gopinath et al. [10].

### ESAT-6 Antibody Optimization for Dispersion of GNP

**Fig. 3:**
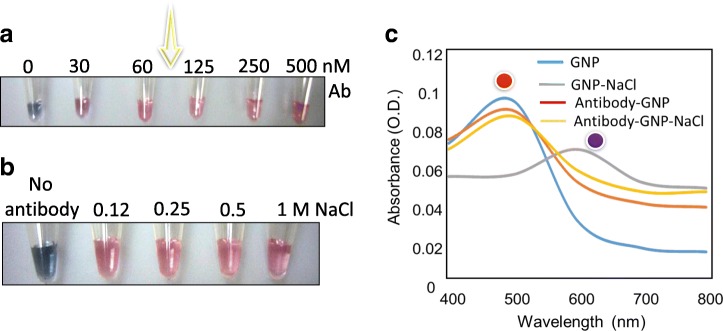
ESAT-6 antibody optimization and stability. Different concentrations of anti-ESAT-6 antibody were tested using 800 mM NaCl. **a** Aggregation or dispersion of GNP with the anti-ESAT-6 antibody (0 to 500 nM); Arrow indicates the transition region for the optimal antibody concentration. **b** Stability of 60 nM of antibody complexed GNP with different NaCl concentrations (0.012 to 1000 mM). **c** Spectra for different complexes. Peaks were indicated with the respective colored spheres

After NaCl optimization, next, we determined the suitable concentration of anti-ESAT-6 antibody to carry out the experiments. Since the sensitivity is highly depending on antibody concentration, it is necessary to desire the suitable concentration of anti-ESAT-6 antibody to induce GNP dispersion. The notion behind is, if the minimal antibody concentration desired, it can be easily complexed upon the addition of the target and there are no free molecules to be attached on the GNP, whereas, if several antibodies are available, when we try to detect the low concentration of ESAT-6, only a small amount of antibodies are complexed. Then, the remaining antibody binds on the GNP surface and induces the stability of GNP, which causes lesser sensitivity. The IgG of the antibody has higher molecular weight (~ 150 kDa), making the free antibodies bind easily on the GNP surface by electrostatic interaction or ionic/hydrogen bonding between the surface-terminated anionic groups on GNP surface [28]. Due to the higher molecular weight, the number of antibodies is lesser, and here, it was balanced with 6 kDa molecular weight ESAT-6, which has more molecules even at a lower concentration. As shown in Fig. 3a, we first added from the lowest concentration of anti-ESAT-6 antibody (30 nM) and increased until the maximum concentration (500 nM) to the GNP. It was shown that 30 nM of anti-ESAT-6 antibody with GNP is in transition of aggregation even with 800 mM NaCl, due to not having enough antibodies available on GNP surface, but from 60 nM of anti-ESAT-6 antibody, the solution has kept its red color owing to the enough number of anti-ESAT-6 antibodies binding on the GNP surface. Until the maximum concentration was tested (500 nM), the GNP color was kept in red color with a high stability. After deciding the suitable concentration to be 60 nM of antibody, to check the stability of anti-ESAT-6 antibody and GNP conjugates, we tried to increase the NaCl concentrations. As shown in Fig. 3b, the prepared antibody GNP conjugate (60 nM antibody with GNP) is very stable even with the addition of 1 M NaCl. The spectrum in Fig. 3c also shows the same result as visual detection; the prepared anti-ESAT-6 antibody GNP conjugates retain its peak at ~ 520 even with a high concentration of NaCl and, at the same time, only GNP leads to aggregate with 800 mM NaCl with the peak maximum at ~ 500 nm.

### ESAT-6 Detection Using Pre-complexed Antibody by Colorimetric Red Spectral Shift and Validation of ESAT-6 Biofouling

**Fig. 4:**
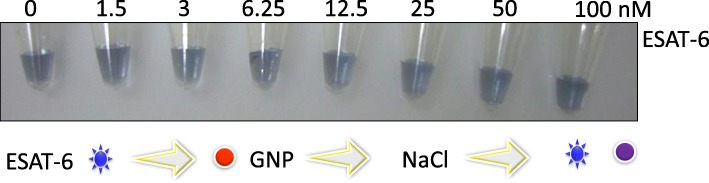
Test for ESAT-6 fouling on GNP surface. Different concentrations of ESAT-6 were mixed with GNP, and after 30 min, 800 mM of NaCl was added. Diagrammatic representation is also shown. The appearance of a blue color indicates the aggregation

**Fig. 5:**
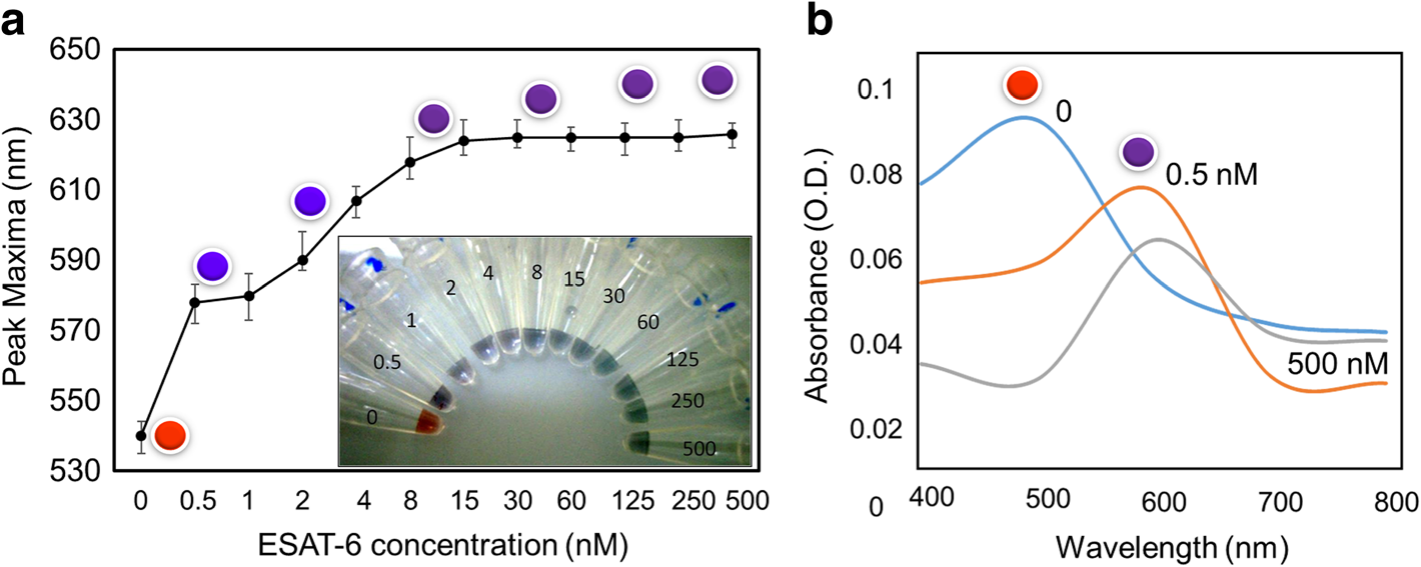
ESAT-6 detection at higher concentrations. **a** Graphical representation for the detection of ESAT-6. Different concentrations of ESAT-6 was mixed with 60 nM of antibody, and after 30 min of incubation, GNP was added. Then 800 mM of NaCl was added to induce the color change. ESAT-6 from 0.5 nM was detected with the clear color change (red to blue). Photographs were taken by the digital camera as in figure inset. **b** Spectral changes at the wavelength from 400 to 800 nm. Peaks were indicated with the respective colored spheres

Since the antibody from 60 nM shows an excellent stability, initially, we tried to detect ESAT-6 complexed with 60 nM of anti-ESAT-6 antibody. ESAT-6 is the smaller molecular weight protein with the size of 6 kDa and well opted for the colorimetric-based assay. Prior to performing the detection experiment, we checked the biofouling of ESAT-6 on the GNP surface; because there is a possibility of ESAT-6 binding with GNP, it may lead to the false positive or false negative. As shown in Fig. 4, in ESAT-6 with the concentration until 100 nM, GNP was not stable at 800 mM of NaCl, indicating that ESAT-6 does not bind electrostatically to the GNP surface. This non-fouling also hinted that the amino acid composition of ESAT-6 is playing an important role in this assay. After this confirmation, we detected ESAT-6 pre-complexed with 60 nM of anti-ESAT-6 antibody. Different concentrations of ESAT-6 were complexed with the constant concentration (60 nM) of antibody and then added to GNP. Finally, 800 mM of NaCl were added to evaluate the aggregation with the red spectral shift. As shown in Fig. 5a (inset), the detection of ESAT-6 from 0.5 nM to 500 nM has confirmed a clear color change with transitions compared to the control (with only 60 nM antibody). The color of the solutions has changed from red to blue even at 0.5 nM and intensified more at 15 nM, and it might be due to the complete saturation with ESAT-6. It seems that no antibody remains to bind with GNP even at 0.5 nM. Referring to the graphical representation of Fig. 5a, 15 to 500 nM of ESAT-6 shows the complete saturation. It is clearly proved from Fig. 5b that the spectrum of control experiment (no ESAT-6 protein) exhibits a nice profile at ~ 500 nm, whereas with ESAT-6 with 0.5 and 500 nM, the spectra were red-shifted. From these results, it was concluded that we can detect ESAT-6 protein from 0.5 nM and still go down to lower concentrations due to obvious blue color appearance.

### Detection Limit of ESAT-6 by the Colorimetric Red Spectral Shift

**Fig. 6:**
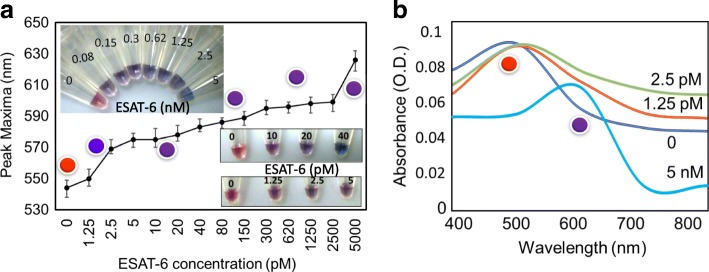
Detection limit of ESAT-6 by the antibody-based colorimetric assay. **a** Graphical representation for the detection of ESAT-6. Different concentrations of ESAT-6 was mixed with 60 nM of antibody, and after 30 min of incubation, GNP was added. Then, 800 mM of NaCl was added to induce the color change. ESAT-6 protein was titrated from 1.25 pM to 5000 pM. Photographs were taken by the digital camera as in figure inset. **b** Spectral changes at the wavelength from 400 to 800 nm. Peaks were indicated with the respective colored spheres

Since 60 nM of anti-ESAT-6 antibody displayed an apparent improvement for the detection of ESAT-6, to find out the limit of detection, we fine-tuned with ESAT-6 further down to the lowest picomolar (1.25 pM to 5000 pM). As shown in Fig. 6a, from 1.25 pM of ESAT-6, there is an initiation of blue color due to the complex formation between ESAT-6 and the antibody. From 2.5 pM, the color of the solution that has changed to blue has intensified and retained the color changes with further concentrations. In the control experiment (without ESAT-6) the color of the solution was appeared to be red, supporting the specificity of the current experiment (Fig. 6a). This result was also confirmed by the spectrum as shown in Fig. 6b. With the control solution, there is no change in the spectrum, but from 1.25 until 5 nM of ESAT-6 protein, the spectra were red-shifted towards 600 nm. At 5 nM of ESAT-6, there was a complete spectral shift observed with peak maxima. Based on these experimental results, we could conclude that the limit of detection of ESAT-6 is around the lowest picomolar (1.25 pM) using antibody ESAT-6 pre-complex.

### Fine-tuning with Specificity

**Fig. 7:**
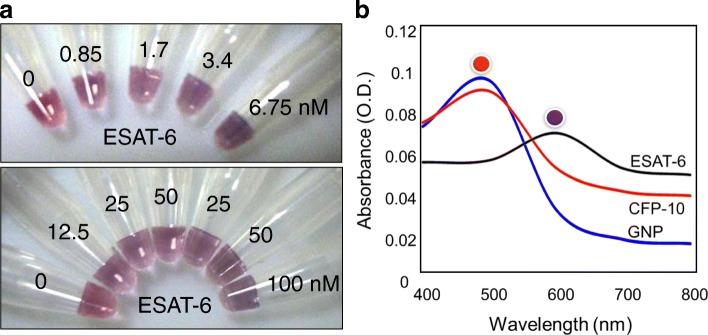
ESAT-6 detection with 75 nM anti-ESAT-6 antibody. **a** Representation for color changes. Different concentrations of ESAT-6 was mixed with 75 nM of antibody, and after 30 min of incubation, GNP was added. Then, 800 mM of NaCl was added to induce the color change. ESAT-6 from 0.85 nM was detected with the clear color change (red to blue). Photographs were taken by a digital camera. **b** Spectral changes at the wavelength from 400 to 800 nm. Peaks were indicated with the respective colored spheres. Specificity assay was carried out using CFP-10 and shown to be a negative control

Since we could detect ESAT-6 with 60 nM of anti-ESAT-6 antibody, next, we tried the same detection with a little high concentration of anti-ESAT-6 antibody to be 75 nM. The obtained results are in Fig. 7a. It has been found that a slight color change occurred using 75 nM pre-complexed antibody at 850 pM ESAT-6. We increased the ESAT-6 concentration to 100 nM, and we could observe the progress in the color changes to blue. With these experiments, the detection limit was noted to be in nanomolar range using 75 nM pre-complexed antibody. Finally, to confirm the specificity of ESAT-6 binding, we also tested the similar assay using pre-complex of anti-ESAT-6 and CFP-10 protein from *M. tuberculosis*. The results are clearly seen that only ESAT-6 has the specificity and CFP-10 can be the appropriate control (Fig. 7b). Overall, from the above results, it was realized that the optimization of antibody concentration is mandatory to improve the sensitivity of the colorimetric assay.

## Conclusions

Tuberculosis (TB) is a major life-threatening disease for human, and the identification of TB at an earlier stage is shown to prevent spreading and to treat the disease. In this study, we choose an early secretory antigenic target (ESAT-6), one of the major proteins in TB. We introduced a single-step antibody-based colorimetric red spectral shift assay using gold nanoparticle, and the detection limit was found to be 1.25 pM. The specificity of this assay was elucidated by using the control protein (CFP-10), and it does not show the color change upon adding GNP. On the other hand, ESAT-6 alone is not bound to GNP. With this evidence, the presence of pre-complexed ESAT-6 and anti-ESAT-6 antibody was demonstrated with the reliability of the colorimetric red spectral shift assay. This strategy is simple and quick to detect similar kinds of analyte as a single step. Further, this assay can be expanded with a small molecule complexed with an appropriate antibody to be suitable for point-of-care detection. This methodology will definitely work with smaller-size proteins and peptides. With larger-size proteins, depending on the amino acid charges, there might be a variation.

## Abbreviations

CFP-10: 10 kDa culture filtrate protein
DNA: Deoxyribose nucleic acid
ESAT-6: 6 kDa early secretory antigenic target
GNP: Gold nanoparticle
NaCl: Sodium chloride
O.D: One optical density
RNA: Ribose nucleic acid
TB: Tuberculosis
UV: Ultraviolet
